# Methoxychlor induces oxidative stress and impairs early embryonic development in pigs

**DOI:** 10.3389/fcell.2023.1325406

**Published:** 2023-12-01

**Authors:** Zhaojun Geng, Yongxun Jin, Fushi Quan, Siyi Huang, Shuming Shi, Bing Hu, Zhichao Chi, Ilkeun Kong, Mingjun Zhang, Xianfeng Yu

**Affiliations:** ^1^ Jilin Provincial Key Laboratory of Animal Model, College of Animal Science, Jilin University, Changchun, China; ^2^ Animal Genome Editing Technology Innovation Center, College of Animal Science, Jilin University, Changchun, China; ^3^ Department of Animal Science, Division of Applied Life Science (BK21 Four), Gyeongsang National University, Jinju, Republic of Korea

**Keywords:** methoxychlor, ROS, embryo, apoptosis, pig

## Abstract

**Introduction:** Methoxychlor (MXC) is an organochlorine pesticide (OCP) that was formerly used worldwide as an insecticide against pests and mosquitoes. However, MXC is not biodegradable and has lipophilic characteristics; thus, it accumulates in organisms and affects reproductive function. MXC, as an estrogenic compound, promotes oxidative stress, induces oxidative stress damage to ovarian follicles, and causes miscarriages and stillbirths in females. In this research endeavor, our primary objective was to explore the ramifications of MXC regarding the developmental processes occurring during the initial stages of embryogenesis in pigs.

**Methods:** In this study, we counted the blastocyst rate of early embryos cultured *in vitro*. We also examined the reactive oxygen species level, glutathione level, mitochondrial membrane potential, mitochondrial copy number and ATP level in four-cell stage embryos. Finally, apoptosis and DNA damage in blastocyst cells, as well as pluripotency-related and apoptosis-related genes in blastocyst cells were detected. The above experiments were used to evaluate the changes of MXC damage on early parthenogenetic embryo development.

**Results and Discussion:** The results showed that early embryos exposed to MXC had a significantly lower cleavage rate, blastocyst rate, hatching rate, and total cell count compared with the control group. It was also of note that MXC not only increased the levels of reactive oxygen species (ROS), but also decreased the mitochondrial membrane potential (ΔΨm) and mitochondrial copy number during the development of early embryos. In addition, after MXC treatment, blastocyst apoptosis and DNA damage were increased, decreased cell proliferation, and the expression of pluripotency-related genes *SOX2, NANOG*, and *OCT4* was down-regulated, while the expression of apoptosis-related genes *BAX/BCL-2* and *Caspase9* was up-regulated. Our results clearly show that MXC can have deleterious effects on the developmental processes of early porcine embryos, establishing the toxicity of MXC to the reproductive system. In addition, the study of this toxic effect may lead to greater concern about pesticide residues in humans and the use of safer pesticides, thus potentially preventing physiological diseases caused by chemical exposure.

## 1 Introduction

Organochlorine pesticides (OCPs) constitute a category of persistent organic pollutants (POPs) that can persist for long periods and be transported over long distances during the degradation process, and can also result in the accumulation of toxic chemical substances in animals and humans (Zhang et al., 2003; Maskaoui et al., 2005; Ali et al., 2014). The accumulation of OCPs in the tissues of organisms positioned at higher trophic levels in the food chain surpasses that observed in organisms occupying lower trophic levels (Snedeker, 2001; El-Shahawi et al., 2010).

OCPs may have adverse effects on the endocrine system and reproduction (Safe, 2004; Toft et al., 2004; Bretveld et al., 2006; Li et al., 2008). China, as a major agricultural country, employed widespread use of dichlorodiphenyltrichloroethane (DDT) between 1940 and 1980. Since 1983, DDT has been banned in China and there has been an increase in the use of methoxychlor (MXC), which is an alternative to DDT. However, MXC has been banned in developed countries, although it is still used in some developing countries. However, in recent years, the toxic effects of MXC and its metabolites have been discovered; as previously reported, *in utero* exposure to high doses of MXC could decrease the levels of testosterone in rat fetuses, consequently leading to malformations of the genital tract in male offspring (Liu et al., 2016a). MXC and its metabolites also inhibit steroidogenesis by decreasing the activity of rat neurosteroidogenic 3α-hydroxysteroid de-hydrogenase (AKR1C14) and retinol dehydrogenase 2 (RDH2) (Mao et al., 2018). MXC causes oxidative stress in adult rat spermatozoa, reduces sperm viability (Aly and Azhar, 2013), and induces testicular cell apoptosis via mitochondrial and FasL-mediated pathways in testicular cells (Vaithinathan et al., 2010).

In addition to its toxicity to the male reproductive system, MXC has an even greater effect on the female reproductive system because it is an insecticide with estrogen-like activity. MXC induces oxidative stress both *in vivo* and *in vitro* within the oocyte, impairing mitochondrial respiration and inducing oxidative damage to proteins and DNA. When exposed to MXC in an aqueous environment, the hepatopancreas and ovarian tissues of the Chinese mitten crab (Eriocheir sinensis) were seriously damaged, with the ovary identified as the target organ of MXC (Cheng et al., 2019). MXC causes a significant increase in the proportion of atretic sinus follicles in adult female mice (Paulose et al., 2012). Within the human environment, the analysis of follicular fluid from 127 infertile women in Hubei Province, China, revealed the presence of seventeen distinct types of OCPs. Remarkably, 13.4% of these detected OCPs featured MXC as the primary compound, with an average concentration of 167.9 ± 33.9 ng/g lipid weight (lw) (Zhu et al., 2015).

The effects of oxidative stress on embryonic development are critical. The addition of several exogenous substances may lead to the generation of reactive oxygen species (ROS) (Ghosh et al., 2012), which will make the oxidant antioxidant level in the cell unbalanced and produce oxidative stress. Cell biomolecules suffer severe oxidative damage and may pass through mitochondria-dependent and mitochondria-independent pathways, causing cell apoptosis (Sinha et al., 2013). Many pesticides have been evaluated for reproductive toxicity risk in laboratory animals (Koç et al., 2009; Bhardwaj and Saraf, 2014). Some of these pesticides can cause oxidative stress in reproductive tissues, leading to ovarian follicle atresia (Symonds et al., 2008; Koç et al., 2009; Banerjee et al., 2014). Adding MXC can induce oxidative stress in mouse ovarian follicles, leading to slow growth and atresia of the follicles (Gupta et al., 2006a). At the same time, MXC has the potential to induce mitochondrial dysfunction and provoke oxidative damage within the mouse ovaries (Gupta et al., 2006b). MXC can induce oxidative stress in rat granulosa cells to induce apoptosis, which leads to follicular atresia. Follicular atresia has a bad influence on female reproductive function (Bhardwaj and Saraf, 2017). The IGF signaling pathway could potentially play a role in mediating MXC-induced disturbances in ovarian function and fertility (Ozden-Akkaya et al., 2017).

The primary cause of human female reproductive toxicity induced by MXC predominantly stems from the induction of oxidative stress, with the ovary being identified as the target organ. However, the impact of MXC on early mammalian embryos is not clear. Therefore, this work aims to directly verify the hypothesis that MXC induces porcine embryo development reduction through the oxidative stress pathway. In this study, the toxic effects of MXC-induced oxidative stress were studied by quantifying ROS levels and mitochondrial membrane potential.

## 2 Materials and methods

All reagents, unless otherwise specified, were procured from Sigma Aldrich (St. Louis, Mo, United States).

### 2.1 Collection of oocytes and cultivation of early embryos

Ovaries were obtained from a slaughterhouse, preserved in a saline solution containing penicillin-streptomycin at 37°C, and promptly transported to the laboratory within a 2-h timeframe by the designated staff. The ovaries were washed two or three times with 0.9% saline at 37°C and then placed into a water bath at 37°C. Follicles of 3–8 mm were obtained by manually pressing the ovaries, creating negative pressure, and inserting a 10 mL syringe with a No. 12 needle on one side. The fluid extracted from the follicles was kept in a 10 mL centrifuge tube and was allowed to precipitate for 10 min. The supernatant was removed using a Pasteur pipette, and the rest of the liquid was cleaned three times with HEPES, by mixing it in the tube and precipitating for 10 min. The oocytes in HEPES were placed in a culture dish. The oocytes with 2-3 layers of cumulus cells were viewed under a stereomicroscope with a hot plate (37°C) and selected using a pipette.

Oocytes were matured *in vitro* by placement in a 4-well dish. To each well, 600 μL of growth medium and 50 oocytes were added and then covered with mineral oil. M199 medium was chosen as the basal culture medium with 10% porcine follicular fluid, 0.01 mg/mL sodium pyruvate, 0.01 μg/mL EGF, also containing PMSG (10 IU/mL) and HCG (10 IU/mL). The oocytes were cultured for a duration of 44 h within a CO_2_ incubator set at 38.5°C, under an atmosphere consisting of 5% CO_2_, 95% air, and a humidity level of 95%.

The cumulus cells of mature oocytes were removed with 0.1% hyaluronidase. The electroactivation of oocytes with the first polar body was performed in order to produce a parthenogenetic embryo. They were kept in 7.5 μg/mL cytochalasin B for 3 h to inhibit the draining of the second polar body. The activated oocytes were transferred to a culture medium (PZM-5) containing different concentrations of MXC. The embryos were then cultivated in a CO_2_ incubator at 38.5°C in an atmosphere of 5% CO_2_, 95% air, and 95% humidity. The oocyte cleavage rate was counted 30 h after activation, and hatching rates were counted after 8 days of incubation. The blastocysts were collected for experiments on day 7.

### 2.2 Treatment with MXC

A stock solution containing 500 μM MXC in DMSO was initially prepared. Subsequently, this solution was further diluted with IVC to yield concentrations of 50 μM, 100 μM, 200 μM, and 500 μM, while maintaining a final DMSO concentration of 1/1,000. These solutions corresponded to four groups, three of which were experimental groups with different concentrations of MXC, and one which was a control group containing 1/1,000 of DMSO. Each experimental group consisted of an average of 50 oocytes. The experiment was replicated three times.

### 2.3 Counting the number of cells in blastocysts

For the purpose of blastocyst cell counting, day 7 blastocysts were gathered from each experimental group. These collected blastocysts were subsequently immersed in a 4% paraformaldehyde solution at room temperature for a duration of 30 min. Following this fixation step, the blastocysts were subjected to three successive washes using PBS-PVA (0.1% PVA). The blastocysts were then transferred to PBS-PVA (0.1% PVA) containing 0.1% TritonX-100 and left to stand for 20 min. Ultimately, the blastocysts underwent an additional three washes with PBS-PVA. Subsequently, they were incubated in a solution containing 5 ng/mL Hoechst33342 for 10 min at room temperature. After the incubation, the blastocysts were carefully transferred onto slides, sealed using an anti-fluorescence quencher, and subjected to visualization under a fluorescence microscope. The counting of cells within the blastocysts was conducted with the aid of ImageJ software.

### 2.4 ROS and GSH assay

Embryos at the 4-cell stage were chosen for further analysis. These embryos underwent a thorough washing process, involving three cycles of rinsing with PBS/PVA. Subsequently, they were subjected to incubation with a ROS detection dye obtained from Invitrogen (NY, United States), along with 10 mM 2′,7′-dichlorofluorescein diacetate (DCFH) sourced from the same manufacturer. After incubation in the dark at 38°C for 30 min, the embryos were imaged using a fluorescence microscope with a digital camera (green fluorescence, UV filters, 490 nm). The embryos were immersed in a GSH dye solution containing 10 mM 4-chloromethyl-6,8-difluoro-7-hydroxycoumarin, which was obtained from Invitrogen (NY, United States). Subsequently, they were incubated at a temperature of 38°C for a duration of 30 min. Images were captured using appropriate filters, including blue fluorescence and UV filters with a wavelength of 370 nm. Subsequently, ImageJ software was employed to quantitatively assess the fluorescence intensity of both ROS and GSH.

### 2.5 Mitochondrial membrane potential assay

The 4-cell stage embryos underwent a thorough washing procedure, involving three rinses with PBS/PVA. Subsequently, they were subjected to incubation with JC-1 dye (5,5′,6,6′-tetrachloro-1,1′,3,3′-tetraethylbenzimidazolocarbocyanine iodide) sourced from Beyotime, China, at a temperature of 38°C for a duration of 30 min. Following this incubation, the embryos were once again washed with PBS/PVA. Images capturing the red and green fluorescence signals were acquired through the use of a fluorescence microscope equipped with a digital camera. The quantification of the ratio between red fluorescence intensity (representing JC-1 aggregates and activated mitochondria) and green fluorescence intensity (corresponding to JC-1 monomers and inactive mitochondria) was conducted using ImageJ software.

### 2.6 TUNEL assay

The day 7 blastocysts were washed three times with PBS/PVA, fixed in 4% paraformaldehyde at room temperature for 30 min, and then washed again three times with PBS/PVA. After washing, the embryos were transferred to a solution of PBS/PVA containing 0.5% Triton X-100 and permeabilized for 30 min, washed again three times with PBS/PVA, and then incubated in TUNEL dye in the dark for 60 min (*In Situ* Cell Death Detection Kit; Roche, Mannheim, Germany). The embryos were stained with 10 ng/mL Hoechst 33342 for a duration of 10 min, followed by three sequential washes with PBS/PVA. Subsequently, they were affixed to microscope slides and subjected to observation using a fluorescence microscope.

### 2.7 EDU cell proliferation stain

Day 6 blastocysts are proliferation stained (38.5°C, 5% CO_2_) using EDU proliferation dye (Beyotime, C0071S). At the end of staining the nucleus was stained using Hoechst 33,342 (5 ng/mL) (Beyotime, C1022) and incubated for 10 min in the dark room, after which the blastocysts were aspirated and washed, sealed with slides and coverslips, photographed using a fluorescence microscope (E179168; Nikon). Amounts of proliferating cells were counted using NIH ImageJ software.

### 2.8 Immunofluorescence staining

Blastocysts were washed 3 times in PBS-PVA and fixed in 4% paraformaldehyde for 30 min after washing. After fixation, they were washed with a solution of PBS containing 0.05% Tween20 (PBST) and treated with 0.1% TritonX-100 at room temperature for 20 min. Blastocysts were washed with PBST three times for 5 min, and immediately after washing, they were treated with 0.1 M, PH = 8 Tris-Hcl for 15 min. Wash again with PBST and close the solution with 2% BSA in PBST for 1 h. Transfer the blastocysts into antibody containing γ-H2AX (2% BSA preparation) overnight at 4 °C. Wash the blastocysts three times with PBST for 5 min and incubate with the secondary antibody (Abbkine, A23420) for 1 h at room temperature. The blastocysts were washed with PBST for 5 min each and incubated with Hoechst 33,342 (5 ng/mL) for 10 min. Finally, the blastocysts were transferred to antifade mounting medium (Abbkine, BMU104-CN) on a slide. Photographs (TIFF format) were taken using a fluorescence microscope (E179168; Nikon), and the NIH ImageJ software was used to analyze and calculate the fluorescence intensity.

### 2.9 Mitochondrial copy number assay

Sixty stage 4-cell stage embryos were collected and DNA was extracted using a DNA extraction kit (QIAGEN, Germany, 80284) according to the instructions. Fluorescence quantification was employed to detect the mitochondrial gene ND1. Simultaneously, the expression levels of the nuclear single-copy gene GCG were measured. To determine the mitochondrial copy number, the relative concentrations of ND1 and GCG were calculated. Specific primer sequences (Table 1) were designed using the National Center of Biotechnology Information (NCBI) database. The specific calculation formula is as follows: N = 2^−Ctmt^/2^−Ctn^

**TABLE 1:** The names of the tested genes, sequences of primers, sizes of PCR products, and accession numbers for RT-qPCR experiments.

Gene name	Sequence	Amplicon size (bpr)	Accession number
*OCT-4*	F: CTA​TGA​CTT​CTG​CGG​AGG​GAT	223	NM_001113060.1
	R: TTT​GAT​GTC​CTG​GGA​CTC​CTC​G		
*SOX-2*	F: ACA​GCA​TGT​CCT​ACT​CGC​AG	278	NM_001123197.1
	R: AGA​GAG​AGG​CAG​TGT​ACC​GT		
*NANOG*	F: TAA​TCA​GAG​CTG​GAG​TAA​CCC​AAC​C	223	NM_001129971.1
	R: TTC​CCC​AGC​AGT​TTC​CAA​GAC		
*ND1*	F: GCC​ACA​TCC​TCA​ATC​TCC​AT	99	NC_000845.1
	R: GAT​TAG​AGG​GTA​GGG​TAT​TGG​TAG		
*ATG5*	F: CAA​AGA​TGT​GCT​TCG​AGA​TGT​G	86	NM_001037152.2
	R: CTG​CCT​CCC​GTT​CAG​TTA​TC		
*ATG7*	F: TGA​ACC​TCA​GCG​AAT​GTA​TGG	98	NM_001190285.1
	R: GTC​CAA​AGT​AGG​GAC​CAA​TCT​C		
*ATG12*	F: CTG​TGG​GAG​ACA​CAC​CTA​TAA​TG	116	NM_001190282.1
	R: CTG​TTC​TGA​AGC​CAC​AAG​TTT​AAG		
*BAX*	F: CAG​CTC​TGA​GCA​GAT​CAT​GAA​GA	194	XM_003127290.5
	R: TCC​TCT​GCA​GCT​CCA​TGT​TA		
*BCL-2*	F: AGC​ATG​CGG​CCT​CTA​TTT​GA	120	XM_021099593.1
	R: GGC​CCG​TGG​ACT​TCA​CTT​AT		
*Caspase9*	F: GGC​CAC​TGC​CTC​ATC​ATC​AA	163	XM_013998997.2
	R: GGAGGTGGCTGGCCTTG		
*GADPH*	F: TCG​GAG​TGA​ACG​GAT​TTG​GC	189	NM_001206359.1
	R: TGA​CAA​GCT​TCC​CGT​TCT​CC		
*GCG*	F: GAA​TCA​ACA​CCA​TCG​GTC​AAA​T	198	NC_010457.5
	R: CTC​CAC​CCA​TAG​AAT​GCC​CAG​T		
*LC3*	F: CAT​GAG​CGA​GTT​GGT​CAA​GAT	138	NM_001170827.1
	R: TCA​TCC​TTC​TCC​TGC​TCA​TAG​A		
*PGC-1α*	F: CCC​ACA​ACT​CCT​CCT​CAT​AAA​G	116	NM_213963.2
	R: TCA​CTG​TAC​CGG​GTC​TTC​T		

F, forward; R, reverse.

N: mtDNA copy number; Ctmt: Ct values for mitochondrial genes; Ctn: Ct values for nuclear single-copy genes.

### 2.10 ATP level detection

ATP levels in 4-cell stage embryos were measured using an ATP assay kit (Beyotime, S0027), using 50 4-cell stage embryos in each group. Measurements were made using ELISA reader (TECAN, Infinite M200, pro).

### 2.11 Quantitative RT-PCR (qRT-PCR)

Blastocysts were collected on day 7, and the extraction of RNA was carried out using an RNA mini kit (Qiagen, Germany, 80284), following the protocol outlined in the provided manual. mRNA was collected from each group of 60 blastocysts. Complementary DNA (cDNA) was synthesized through the reverse transcription of mRNA, employing a reverse transcription kit (Tiangen, China). Quantitative real-time PCR was carried out utilizing a fluorescent quantitative assay kit (Tiangen, China). The PCR protocol involved an initial heating step at 95°C for 5 min, followed by 40 cycles of denaturation at 95°C for 15 s, annealing at 60°C for 25 s, and extension at 72°C for 20 s. The final extension steps were performed at 72°C. The target genes were *NANOG*, *OCT4*, *SOX2*, *BCL-2*, *BAX* and *Caspase9*. The primer sequences utilized in this investigation are detailed in Table 1.

### 2.12 Statistical analysis

In this study, all experiments were independently repeated three times. Statistical analysis was performed using GraphPad Prism 9 software (GraphPad Software, San Diego, CA, United States). The assessment of fluorescence intensities was conducted using ImageJ software (National Institutes of Health in Bethesda, MD, United States). The normality of the data was assessed using the Shapiro-Wilk test, and outliers exceeding ±2 times the standard error of the mean (SEM) were excluded. Experimental data were compared using Student’s t-test, with each group compared to the control group. *p* < 0.05 is considered statistically significant and the results were presented as mean ± SEM.

## 3 Results

### 3.1 MXC inhibited early embryonic development in pigs

**FIGURE 1:**
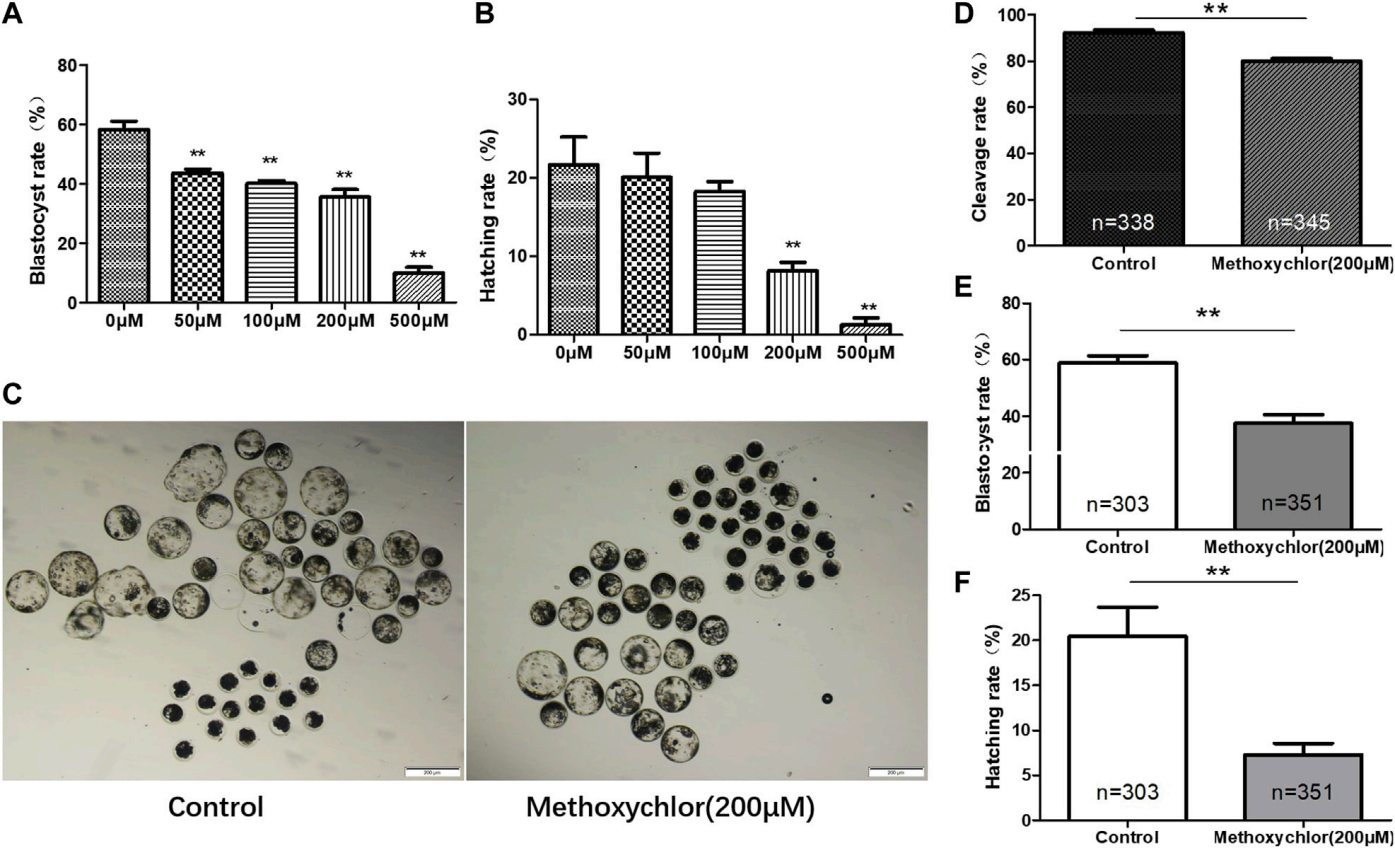
Impact of MXC on parthenogenetic pig embryos. The data from each group were compared only to the control group. **(A)** The blastocyst rates of early pig embryos at different concentrations of MXC (magnified 40 times, scale bar = 200 µm). **(B)** The hatching rates of early pig embryos exposed to different concentrations of MXC. **(C)** Blastocyst formation in the control group and the 200 μM MXC group. **(D)** Cleavage rates in the control group and the 200 μM MXC group. **(E)** Blastocyst rate in the control group and the 200 μM MXC group. **(F)** Hatching rate in the control group and the 200 μM MXC group. They were expressed as mean ± SEM, significance difference is denoted by **p < 0.01.

After parthenogenetic activation, the oocytes were placed in IVC medium containing different concentrations of MXC. We examined the blastocyst rates and the hatching rates of embryos exposed to different concentrations of MXC (control group, 50 μM, 100 μM, 200 μM, and 500 μM MXC). The blastocyst rates were significantly different among groups (58.25% ± 2.83%, 43.65% ± 1.28%, 40.10% ± 1.01%, 35.49% ± 2.57%, 9.94% ± 1.95%, *p* < 0.05, Figure 1A). Compared with other concentrations, 200 μM and 500 μM MXC significantly reduced the hatching rate (21.68% ± 3.57%, 20.12% ± 3.07%, 18.29% ± 1.28%, 8.18% ± 1.09%, 1.31% ± 0.85%, *p* < 0.05, Figure 1B). Compared with those treated with 200 μM MXC, almost no early pig embryos treated with 500 μM MXC demonstrated development. Hence, 200 μM MXC was selected for the subsequent experiments (Figure 1C). The exposure of parthenogenetic embryos to 200 μM MXC significantly reduced the cleavage rate to lower than in the control group (92.30 ± 1.06% vs. 80.11% ± 1.02%, *p* < 0.05, Figure 1D). The rate of blastocyst was notably lower when compared to that observed in the control group (58.93 ± 2.49% vs. 37.57% ± 3.00%, *p* < 0.05, Figure 1E). The hatching rate was also significantly lower than that in the control group (20.44 ± 3.26% vs. 7.29% ± 1.27%, *p* < 0.05, Figure 1F). Therefore, 200 μM MXC was shown to result in significant inhibition of early embryonic development.

### 3.2 MXC induced oxidative stress and increased ROS levels in embryos

**FIGURE 2:**
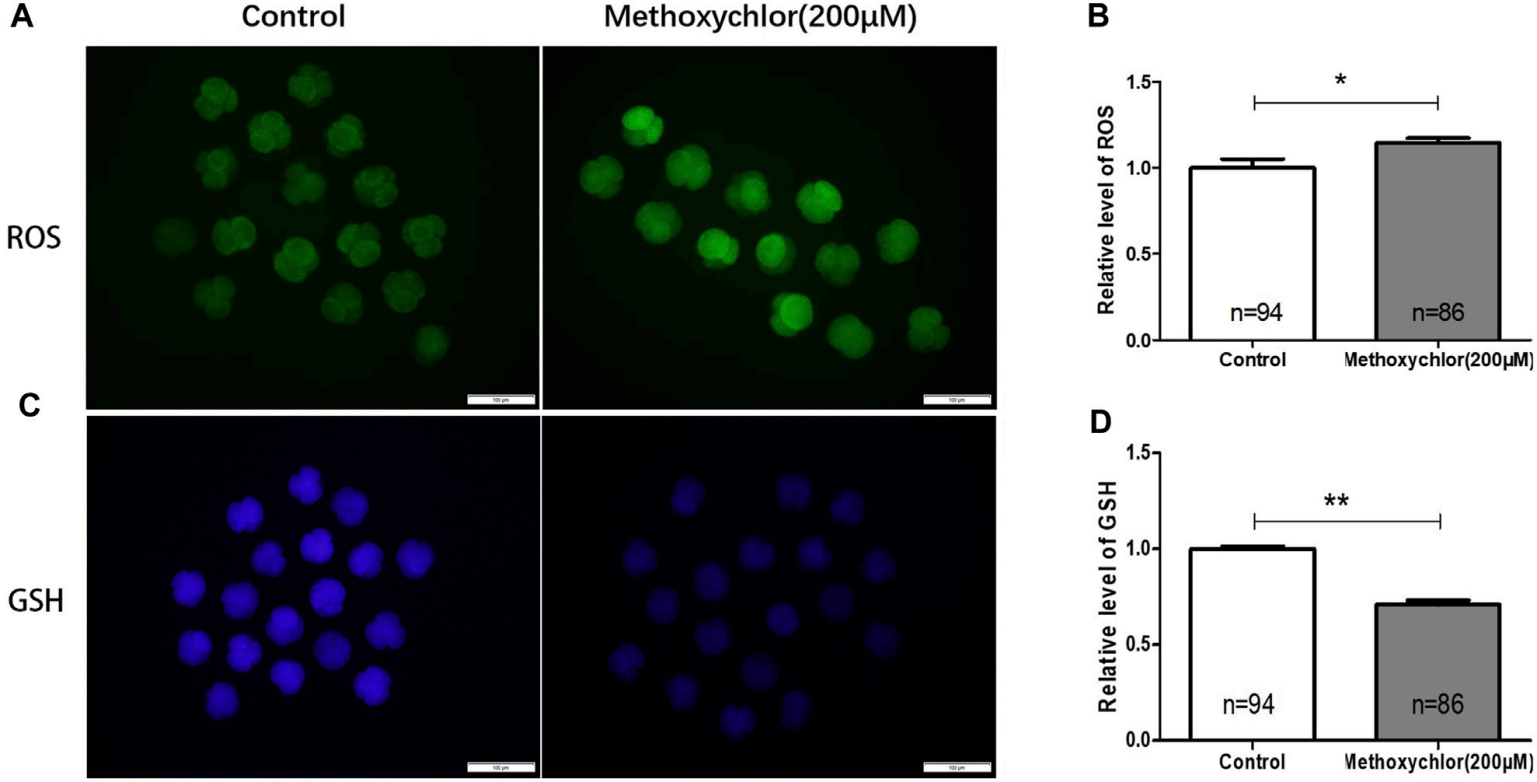
The influence of MXC on the levels of ROS and GSH in early pig embryos. **(A)** The ROS fluorescence intensity in 4-cell stage early embryos (magnified 100 times, scale bar = 100 µm). **(B)** The relative levels of ROS in 4-cell stage embryos. A significant difference is denoted by * (*p* < 0.05). **(C)** The GSH fluorescence intensity in 4-cell stage early embryos (magnified 100 times, scale bar = 100 µm). **(D)** The relative levels of GSH in 4-cell stage embryos. A highly significant difference is denoted by **(*p* < 0.01). They were expressed as mean ± SEM, significant difference is denoted by *(*p* < 0.05), **(*p* < 0.01).

Changes in reactive oxygen species (ROS) can have a significant impact on oocytes, and an excess of ROS can lead to damage in oocytes (Agarwal and Allamaneni, 2004). Glutathione (GSH), an essential antioxidant molecule involved in the removal of ROS, has been found to protect oocytes from oxidative stress (Barros et al., 2019). The excessive production of ROS can result in oxidative damage to various macromolecules, including lipids, proteins, and DNA. To determine if MXC induced ROS production, the formation of ROS in 4-cell stage embryos treated with MXC was examined using dichlorodihydrofluorescein diacetate (DCFH-DA). As illustrated in Figure 2, the relative fluorescence intensity of ROS in embryos subjected to MXC treatment exhibited a notable increase compared to that of the control group at the 4-cell stage. (1.00 ± 0.05, *n* = 94 vs. 1.15 ± 0.03, *n* = 86, *p* < 0.01, Figures 2A, B). In the subsequent analysis of fluorescence intensity, the glutathione (GSH) fluorescence intensity in embryos treated with MXC was lower than that of the control group (1.00 ± 0.01, *n* = 94 vs. 0.71 ± 0.02, *n* = 86, *p* < 0.05, Figures 2C, D). The results showed that MXC could increase oxidative stress in early pig embryos.

### 3.3 Impact of MXC on mitochondrial function during early embryonic development in pigs

**FIGURE 3:**
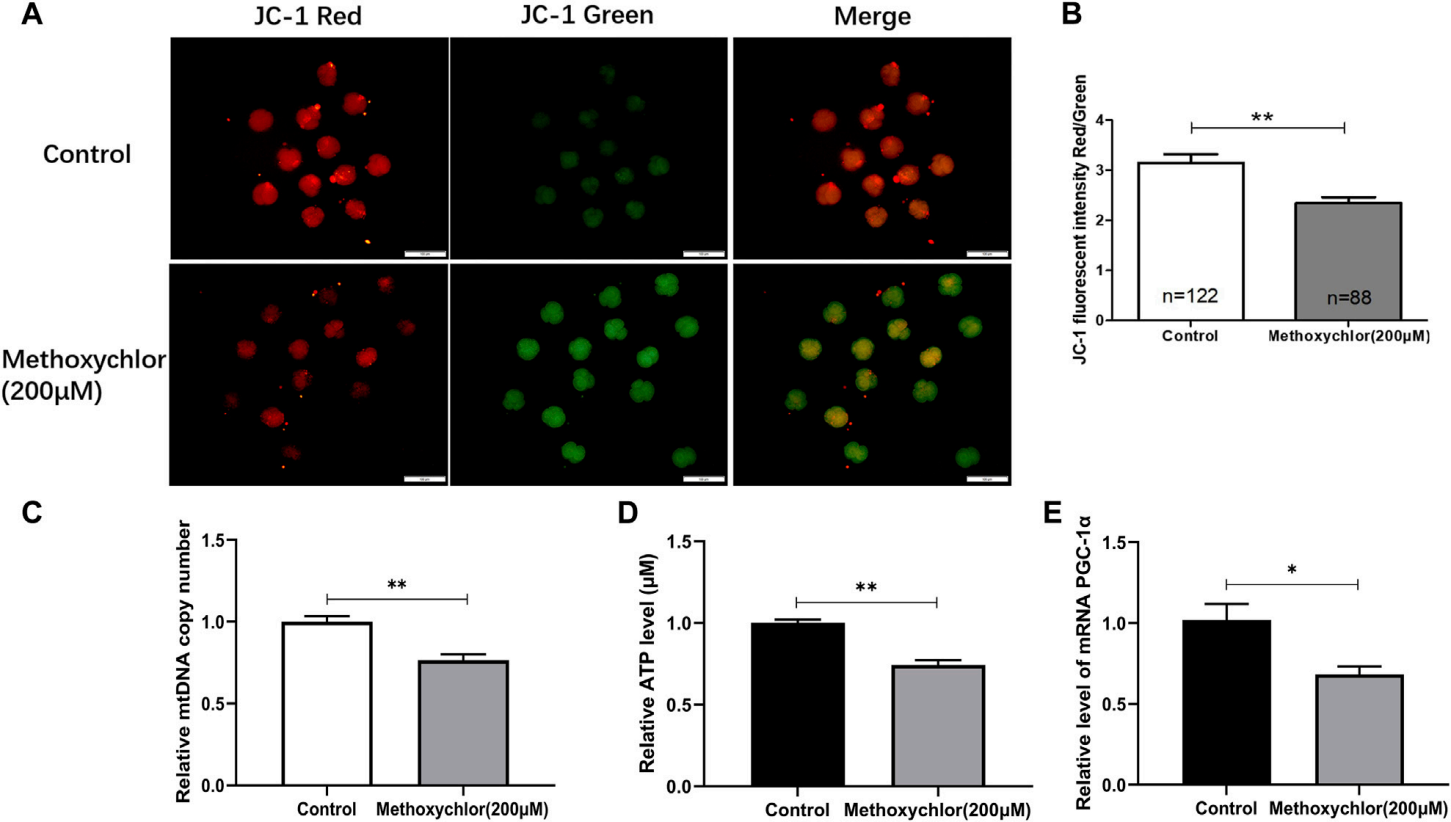
MXC may induce mitochondrial dysfunction in 4-cell stage early embryos. **(A)** Embryos at the 4-cell stage subjected to JC-1 staining (magnified 100 times, scale bar = 100 µm). **(B)** The relative levels of JC-1 fluorescence intensity. **(C)** Relative copy number of mitochondria in 4-cell stage embryos (*n* = 80). **(D)** Relative ATP level in 4-cell stage embryos (*n* = 50). **(E)** Relative expression of mitochondria-related gene *PGC-1α* in 4-cell stage embryos. They were expressed as mean ± SEM, significant is denoted by *(*p* < 0.05), **(*p* < 0.01).

Mitochondria provide the energy required for early embryonic development, and are thus essential. The mitochondrial membrane potential stands as one of the foremost critical indicators used to assess mitochondrial function. To clarify the mechanism by which MXC affected early embryonic development in pigs, the ΔΨm of the 4-cell stage embryos was measured. ΔΨm is shown in Figure 3. As revealed through JC-1 staining, it became evident that embryos exposed to 200 mM MXC exhibited a significantly reduced ΔΨm in comparison to the control group. This decline in ΔΨm signifies a disturbance in mitochondrial function (3.15 ± 0.17, *n* = 122 vs. 2.35 ± 0.12, *n* = 88, *p* < 0.01, Figures 3A, B). We then examined the mitochondrial copy number in 4-cell stage embryos and found that the mitochondrial copy number was significantly reduced in the MXC-treated group (1.00 ± 0.035 vs. 0.764 ± 0.036, *p* < 0.01, Figure 3C). Mitochondria are an important site of ATP synthesis, and a large amount of ATP is required for embryo development. Our assay of ATP levels in embryos at the 4-cell stage showed that MXC reduced ATP content during embryo development (1.00 ± 0.021, vs. 0.743 ± 0.030, *n* = 50, *p* < 0.01, Figure 3D). The expression of the mitochondria-related gene *PGC-1α* was examined and found to be significantly reduced by MXC treatment (Figure 3E). The above results suggest that MXC caused mitochondrial dysfunction during early embryonic development.

### 3.4 Impact of MXC on apoptosis in early pig embryos

**FIGURE 4:**
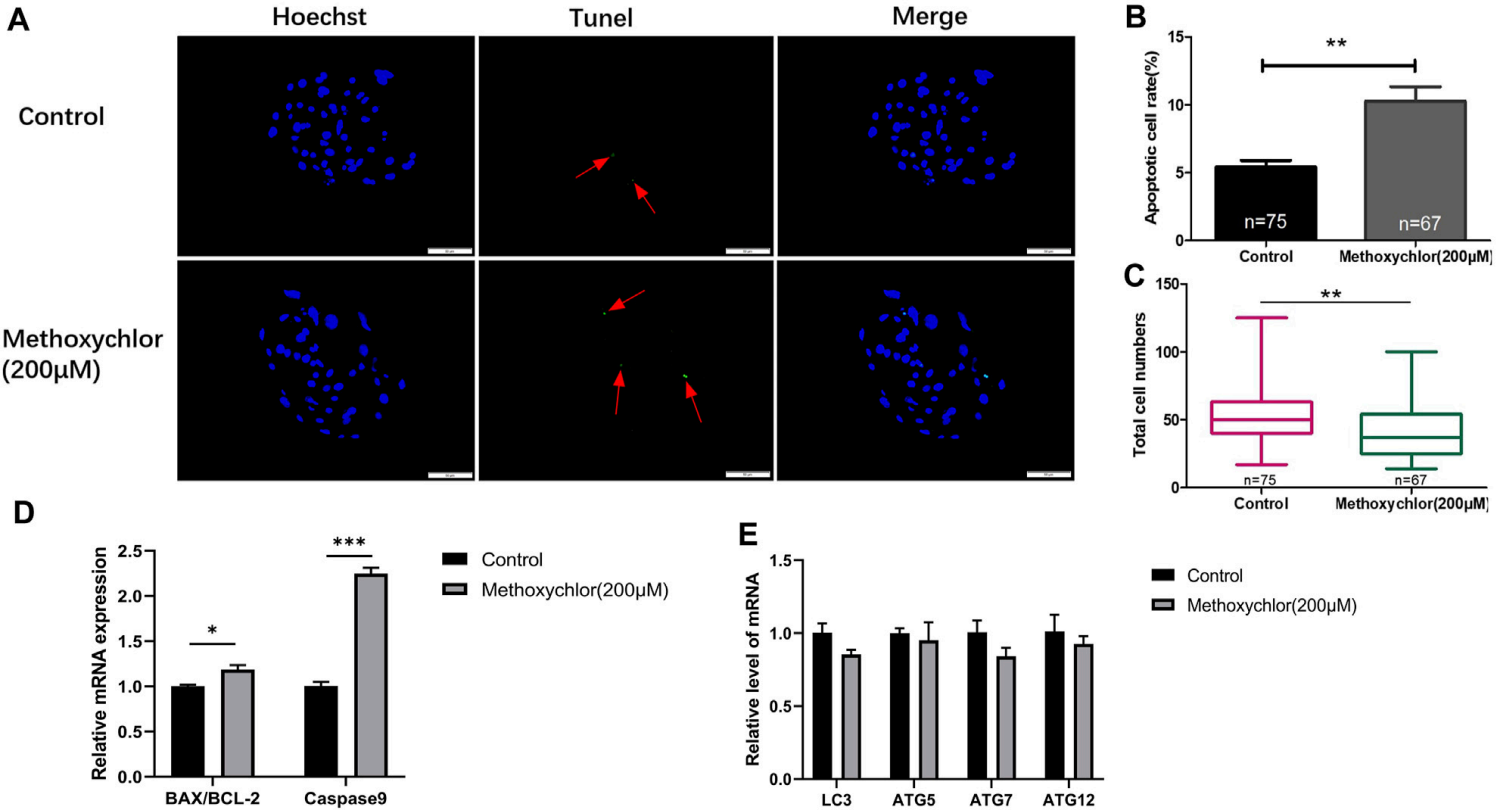
Impact of MXC on apoptosis. **(A)** Blastocysts on Day 6 dyed stained with Hoechst 33342 and TUNEL (magnified 200 times, scale bar = 50 µm). The arrows point to the nucleus that were stained positively for apoptosis. **(B)** The apoptotic blastocyst cell rates in the control group and the MXC group. **(C)** The total cell numbers of the control group and the MXC group on Day 6. **(D)** Relative expression of apoptosis-related genes *BAX/BCL-2* and *Caspase9*. **(E)** Relative expression of autophagy-related gene expression *LC3*, *ATG5*, *ATG7* and *ATG12*. They were expressed as mean ± SEM, significant is denoted by *(*p* < 0.05), **(*p* < 0.01), ***(*p* < 0.001).

To confirm the negative impact of MXC on the embryos, the apoptosis of MXC-treated blastocyst cells was examined using the TUNEL assay. As shown in Figure 4, there was a greater extent of cellular apoptosis in the group treated with MXC (5.4 ± 0.48, *n* = 75 vs. 10.26 ± 1.07, *n* = 67, *p* < 0.01, Figures 4A, B). At the same time, the number of blastocyst cell was also reduced (53.61 ± 2.43, *n* = 75 vs. 40.96 ± 2.39, *n* = 67, *p* < 0.01, Figure 4C). We also examined the expression of apoptosis-related genes *BAX*, *BCL-2* and *Caspase9* in blastocyst cells and found that the expression of apoptosis-related genes was increased in the MXC-treated group (Figure 4D). Autophagy plays a role in cells to remove damaged cells and maintain cellular homeostasis and embryonic development (Mizushima and Komatsu, 2011). We subsequently examined the expression of autophagy-related genes in blastocyst cells to investigate whether the increase in the level of apoptosis in blastocyst cells led to an increase in the level of autophagy, and the results showed that no significant changes in the expression of autophagy-related genes were detected in blastocyst cells after MXC treatment (Figure 4E). The results show that treating embryos with MXC increases the level of apoptosis but autophagy is not activated.

### 3.5 Impact of MXC on DNA damage in early porcine embryos

**FIGURE 5:**
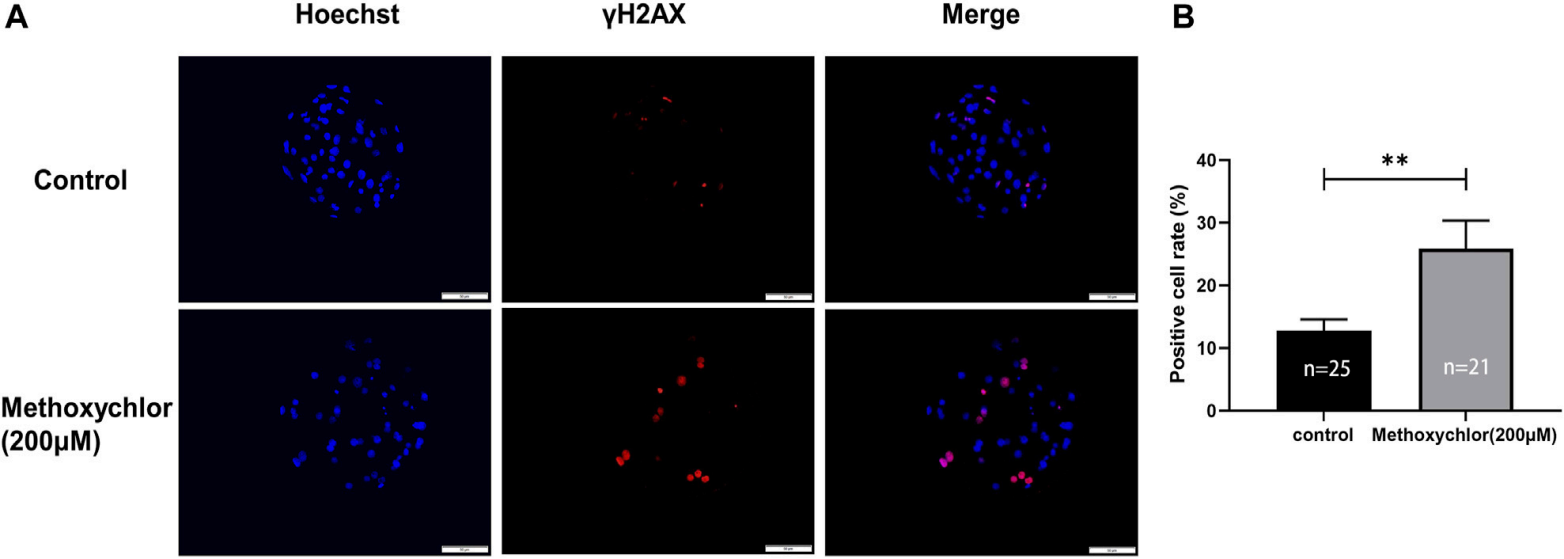
Impact of MXC on DNA damage. **(A)** Representative fluorescence images of day seven blastocyst γH2AX immunofluorescence staining (magnified 200 times, scale bar = 50 µm). **(B)** Ratio of the number of cells producing DNA breaks in blastocyst cells. They were expressed as mean ± SEM, significant differences are indicated by **(*p* < 0.01).

To determine the effect of MXC on DNA damage in blastocyst cells. We examined DNA damage in blastocyst cells by immunofluorescence staining using the DNA break marker γH2AX. We found a significant increase in the number of DNA breaks produced by cells in the MXC-treated group (12.79% ± 1.79%, *n* = 25 vs. 25.89% ± 4.47%, *n* = 27, *p* < 0.01, Figures 5A, B). The results showed that MXC increased DNA damage in blastocyst cells.

### 3.6 Impact of MXC on the proliferative capacity of porcine embryos

**FIGURE 6:**
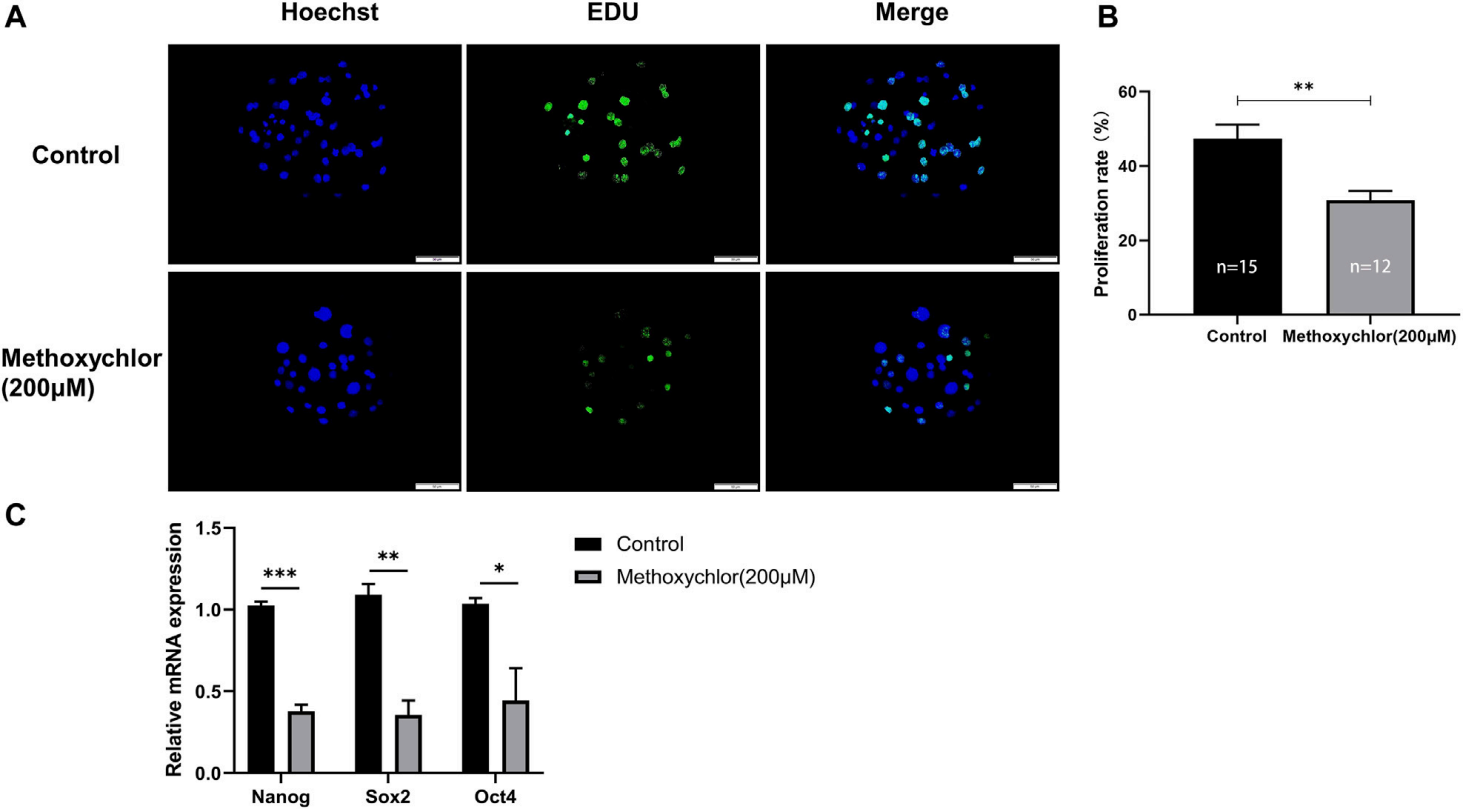
Impact of MXC on the proliferative capacity of embryos. **(A)** Representative fluorescence images of day six blastocyst EDU staining (magnified 200 times, scale bar = 50 µm). **(B)** Proliferation rate of blastocyst cells in each group. **(C)** Pluripotency-related gene expression in day seven blastocysts in control and MXC (200 μM) group (*n* = 60 per group). They were expressed as mean ± SEM, significant difference is denoted by *(*p* < 0.05), **(*p* < 0.01), ***(*p* < 0.001).

To determine whether MXC affects the proliferative capacity of early parthenogenetic embryos, we have stained day six blastocysts for EDU cell proliferation. In the MXC group, a significant reduction in the number of proliferating cells was observed compared to the control group (47.334 ± 8.474, *n* = 15 vs. 30.777 ± 6.096, *n* = 12, *p* < 0.01, Figures 6A, B). We then examined day seven blastocysts the expression of totipotency-related genes *NANOG*, *SOX2* and *OCT4*. The results showed that MXC treatment affected the expression of pluripotency genes in early embryos (Figure 6C). The results indicated that MXC reduced the proliferative capacity of early parthenogenetic embryos and affected the quality of embryonic development.

## 4 Discussion

In the past few years, food security and environmental pollution have emerged as two of the most popular research topics related to human health and are future challenges facing the Earth. Aside from workers in the agricultural industry, the general public may also be exposed to OCPs through various routes, including food and water. OCPs have been identified in various environmental compartments such as air, soil, water, and sediment, as well as in commonly consumed items such as food, vegetables, fish, and poultry. Furthermore, these compounds have also been found in biological samples including blood, adipose tissue, breastmilk, and cord blood within the general population in China (Nakata et al., 2002; Qiu et al., 2004; Nakata et al., 2005; Poon et al., 2005; Yang et al., 2005; Yang et al., 2006; Zhou et al., 2006; Wang et al., 2007; Tao et al., 2009; Luo et al., 2016). MXC-induced oxidative changes also occur in other reproductive organs (Gangadharan et al., 2001; Latchoumycandane et al., 2002). We studied the effects of MXC on early pig embryos using methods described in a paper that investigated the impact of MXC on oxidative stress in oocytes and their meiosis in mice (Liu et al., 2016b). We found that MXC increased the level of superoxide free radicals and other ROS in early pig embryos, increased the percentage of apoptotic blastocysts and DNA damage, and decreased the mitochondrial membrane potential, mitochondrial copy number, cell proliferation capacity, total number of blastocysts, and the blastocyst rate. Therefore, it can be concluded that MXC adversely affects the development of early pig embryos.

The persistence of MXC as an environmental contaminant in the environment affects embryonic development. Blastocyst rate and hatching rate are important reference standards for judging the quality of *in vitro* embryo development. MXC inhibits embryo development and reduces the number of cells in blastocysts (Amstislavsky et al., 2003). This is consistent with the results of the present study in which MXC exposure reduced the rate of blastocysts in parthenogenesis, and the number of blastocyst cells and their hatching rate.

Free radicals are generated as part of routine metabolic processes, and they play integral roles in normal cellular functions, including cell signal transduction, gene expression, and the regulation of cell apoptosis (Ghosh and Myers, 1998). However, in addition to the endogenous free radicals produced by normal metabolism, there are also exogenous sources. The primary external origins of free radicals include smoking, exposure to air pollution, ultraviolet and ionizing radiation, as well as suboptimal food quality (Valko et al., 2007). GSH is an important antioxidant molecule involved in the elimination of ROS and has been shown to reduce oxidative stress in cells (Barros et al., 2019). MXC caused a significant increase in ROS levels in mouse oocytes, contributing to oxidative stress (Liu et al., 2016b). This is consistent with the results in this study, where MXC led to an overproduction of ROS and a decrease in GSH content during early embryonic development, which exerted oxidative stress on early embryos. We believe that this is the main cause of MXC-induced developmental damage to early pig embryos.

Mitochondria are important organelles that provide ATP for most cellular energy-demanding processes through the oxidative phosphorylation pathway in the early embryo (Niu et al., 2020). Mitochondria are also the main source of ROS production, and the electron transport chain in mitochondrial respiration is accompanied by ROS production. The number of mitochondria in an embryo is one of the markers of embryo quality (Kobayashi et al., 2020). Mitochondrial membrane potential provides the foundation for the respiration function of mitochondria, converting ADP into ATP under the action of enzymes. The depolarization of ΔΨm can disrupt the transfer of electrons to oxygen receptors, leading to the overproduction of ROS (Lenaz, 2001; St-Pierre et al., 2002; Djavaheri-Mergny et al., 2003; Pelicano et al., 2003; Hagen et al., 2004), and excessive ROS will induce cell apoptosis (Valko et al., 2007). Due to the high reactivity of free radicals, the excess production of free radicals can induce oxidative stress damage to macromolecules (such as lipids, proteins and DNA) by affecting their functions (Goutzourelas et al., 2018). It has been shown that MXC exposure increases hydrogen peroxide production in rat brain cells, decreased mitochondrial membrane potential, and inhibits mitochondrial respiration in mice (Schuh et al., 2005). MXC also causes excessive production of ROS in the mouse ovary, leading to mitochondrial dysfunction in the ovary and thus reproductive dysfunction (Gupta et al., 2006b). The decrease in mitochondrial copy number is also evidence of a decrease in embryo quality (Gil et al., 2012). This is consistent with the findings of this study that exposure to MXC may lead to the depolarization of mitochondrial membrane potential, reduced mitochondrial copy number, decreased ATP levels, causing the overproduction of ROS.

The delicate equilibrium between ROS levels and endogenous antioxidants, when disrupted, can culminate in oxidative stress and, in more severe scenarios, trigger apoptosis (Soga et al., 2012). Mitochondria are involved in the regulation of cell death pathways (Green and Reed, 1998; Kaufmann et al., 2000) and occupy a pivotal role in the integration and transmission of cell death signals, encompassing factors like oxidative stress and DNA damage (Green and Kroemer, 2004). Mitochondria have important functions in apoptosis, and changes in the mitochondrial membrane potential are a critical mediator of apoptosis induction (Liu et al., 2000). It has been shown that MXC significantly increases the rate of apoptosis in mouse preimplantation embryos and affects embryonic development (Amstislavsky et al., 2003). The TUNEL assay shows that compared with the control group, the blastocyst rate of the experimental group exposed to MXC was decreased, while the extent of apoptosis was increased. The results of the detection of DNA damage in blastocysts showed that MXC resulted in increased DNA damage in embryos. The data from this study show that the exposure of early pig embryos to MXC induced apoptosis and increases DNA damage.

The proliferation and orderly differentiation of embryonic cells play crucial role in mammalian embryonic development, and the expression of pluripotency genes is closely related to the quality of early embryonic development (Suwinska and Ciemerych, 2011). Our results suggest that MXC does have a reducing effect on the proliferative capacity of early porcine embryos. Previous studies have shown that MXC can reduce embryo totipotent gene expression, and the oxidative stress induced by MXC may cause changes in Bcl-2 family members (Gupta et al., 2006a). This in turn leads to the expression of Caspase9, which induces apoptosis. We examined gene expression in day 7 parthenogenetic blastocysts. The qRT-PCR result revealed a notable elevation in the expression of blastocyst apoptosis-related genes, namely, *BAX*/*BCL-2* and *Caspase9*, within the experimental group. Conversely, the expression levels of pluripotency genes, including *NANOG*, *Oct4*, and *Sox2*, displayed a discernible reduction in the experimental group. Therefore, these results show that the exposure of early pig embryos to MXC may reduce their development potential.

Using pig *in vitro* fertilized (IVF) embryos to study the effects of MXC on early-stage embryos is more meaningful. While IVF embryos and parthenogenetic embryos (PA) show similar patterns in mitochondrial activity during development (Du et al., 2021), their gene expression profiles differ at various time points (Razmi et al., 2023). However, compared to cattle and mice, pigs exhibit the unique characteristic of polyspermy during IVF, where multiple sperm can fertilize a single oocyte, leading to different gene expression patterns in embryos that result from polyspermy (Funahashi, 2003). This can introduce potential errors in experiments.

Parthenogenetic embryos, on the other hand, are widely used in early embryonic development research due to their stability and ease of acquisition (Chen et al., 2023). The focus of this study is the impact of the environmental pollutant MXC on early embryo development, and therefore, using more stable PA embryos for experimentation is justified. Given the distinct developmental patterns of IVF embryos and PA embryos, we plan to investigate the effects of MXC on IVF embryo development and *in vivo* embryo development in future experiments.

In summary, our results have unequivocally demonstrated that MXC elicits detrimental impacts on the developmental processes of early pig embryos, thereby reinforcing MXC’s toxicity to the reproductive system. In addition, research on this toxic effect may cause humans to pay more attention to pesticide residues, use safer pesticides, thus potentially preventing the physiological diseases caused by chemical exposure.

## 5 Conclusion

Our results show that MXC has reproductive toxicity, which impedes the development of early embryos by increasing ROS, decreasing mitochondrial function, promoting embryonic apoptosis and DNA damage.

## Data Availability

The original contributions presented in the study are included in the article/Supplementary material, further inquiries can be directed to the corresponding authors.
